# Sophisticated Communication in the Brazilian Torrent Frog *Hylodes japi*

**DOI:** 10.1371/journal.pone.0145444

**Published:** 2016-01-13

**Authors:** Fábio P. de Sá, Juliana Zina, Célio F. B. Haddad

**Affiliations:** 1 Laboratório de Herpetologia, Departamento de Zoologia, Universidade Estadual Paulista, Rio Claro, São Paulo, Brazil; 2 Departamento de Ciências Biológicas, Universidade Estadual do Sudoeste da Bahia, Jequié, Bahia, Brazil; University of Sao Paulo, BRAZIL

## Abstract

Intraspecific communication in frogs plays an important role in the recognition of conspecifics in general and of potential rivals or mates in particular and therefore with relevant consequences for pre-zygotic reproductive isolation. We investigate intraspecific communication in *Hylodes japi*, an endemic Brazilian torrent frog with territorial males and an elaborate courtship behavior. We describe its repertoire of acoustic signals as well as one of the most complex repertoires of visual displays known in anurans, including five new visual displays. Previously unknown in frogs, we also describe a bimodal inter-sexual communication system where the female stimulates the male to emit a courtship call. As another novelty for frogs, we show that in addition to choosing which limb to signal with, males choose which of their two vocal sacs will be used for visual signaling. We explain how and why this is accomplished. Control of inflation also provides additional evidence that vocal sac movement and color must be important for visual communication, even while producing sound. Through the current knowledge on visual signaling in Neotropical torrent frogs (i.e. hylodids), we discuss and highlight the behavioral diversity in the family Hylodidae. Our findings indicate that communication in species of *Hylodes* is undoubtedly more sophisticated than we expected and that visual communication in anurans is more widespread than previously thought. This is especially true in tropical regions, most likely due to the higher number of species and phylogenetic groups and/or to ecological factors, such as higher microhabitat diversity.

## Introduction

Communication assumes a sender transferring a message to a receiver via codified signals that both are able to understand [1, 2]. Acoustic communication has been considered the main intraspecific communication mode among most frog species and historically has been studied more than any other mode [3, 4]. Research focusing on visual communication has become more common lately, revealing that visual signaling is indeed relevant for several diurnal and nocturnal frog families (e.g., [5–8]). Multimodal communication employs two or more sensory modalities in combination to compose complex signals. Multimodal signaling is known in several animal groups, such as wolf spiders [9, 10], fishes [11, 12], squirrels [13, 14], and birds [15, 16]. Studies on multimodal signaling have made considerable progresses towards a more complete understanding of intraspecific frog communication (e.g., [17–20]).

The latest review of visual communication in anurans showed great communication complexity in frogs [2]. The diversity of visual display repertoires of frogs is variable among families, suggesting several cases of independent evolution (see [2, 21]). Plausibly, evolution of visual communication in frogs is favored by particular ecological factors. Visual display repertoires tend to be complex in diurnal species that breed in noisy environments, such as *Hylodes asper* from Brazil (family Hylodidae; e.g., [6]), *Micrixalus saxicola* from India (family Micrixalidae; e.g., [22]), and *Staurois parvus* from Borneo (family Ranidae; e.g., [23]).

The most recent review of visual communication in anurans [2] found some groups of species to be crucial to understanding the evolution of visual communication in anurans. Among these groups the authors included the genus *Hylodes*, a Brazilian endemic whose species dwell in fast-flowing streams. Since that compilation fourteen years ago, many new records on visual displays have been published for several species of frogs, including hylodids such as *Hylodes cardosoi* [24] and *Hylodes phyllodes* [21]. Species of *Hylodes* are excellent models for investigating communication because they usually call and exhibit extraordinary visual display repertoires associated with various behavioral contexts (e.g., [6, 21, 24, 25]). For these reasons, species of *Hylodes* deserve to be studied in more detail, particularly concerning potential multimodal signals.

Since it is clear that studies involving species of *Hylodes* are critical to better understanding frog communication, we investigate how the Brazilian torrent frog *Hylodes japi* communicates intraspecifically. *Hylodes japi* was recently described as endemic to the montane Atlantic forest of Serra do Japi, in southeastern Brazil [26]. This small frog is mainly diurnal, however it may also exhibit crepuscular and nocturnal reproductive activity [26, 27]. The species is rheophilic, has territorial males and an elaborate courtship behavior, as do other members of the family Hylodidae. The reproductive biology of *H*. *japi* is associated with fast-flowing montane streams where males construct underwater chambers for egg deposition; the tadpoles are exotrophic [26]. Our goals were to: (1) characterize visual displays and acoustic signals performed by males and females of *H*. *japi*, and to identify their respective roles and associated behavioral contexts; (2) investigate communication during their elaborate courtship ritual, including analyzing potential multimodal compositions and their roles; and (3) characterize the ways by which a sender male controls signal emission according to the position of the receiver male. We discuss the behavioral diversity in the family Hylodidae in light of current knowledge on visual signaling in Neotropical torrent frogs.

## Materials and Methods

We conducted our study in the biological reserve of Serra do Japi, Jundiaí county, state of São Paulo, southeastern Brazil, an ecotonal region mainly covered by seasonal semideciduous mesophytic forests [28]. It is an important remnant of the threatened Atlantic Forest, still supporting an exuberant diversity of flora and fauna [29, 30]. We concentrated our field studies in the Ribeirão Ermida, a fast-flowing stream in the northern part of the reserve (23°13’S, 46°58’W; 880 m elev.). Our research was carried out in strict accordance with the recommendations in the Guide for the Care and Use of Laboratory Animals of the National Institutes of Health. Our field procedures were previously approved and permitted by Secretaria Municipal de Planejamento e Meio Ambiente (SMPMA) de Jundiaí (São Paulo, Brazil), Ethics Committee on Animal Use of Universidade Estadual Paulista (UNESP), Rio Claro, São Paulo, Brazil (license number 3501/20), and Instituto Chico Mendes de Conservação da Biodiversidade/Instituto Brasileiro do Meio Ambiente e dos Recursos Naturais Renováveis (ICMBio/IBAMA; license numbers 14846–3 and 25966–2).

We made 17 visits to the field (the first being a pilot study), over a period of 15 consecutive months during 2011 and 2012, for a total of 93 days in the field and 206 h directly collecting data. We obtained behavioral data with the use of focal animal and all-occurrence sampling methods [31, 32]. In the field, we made direct observations and filmed male and female behaviors with the use of a Sony camcorder (HDR-XR550V). We followed a previously proposed approach [2] to include behavioral events as intraspecific visual displays into our analyses. A behavioral event was considered to be a visual display when it met the following criteria: (1) may provide a visual cue during an intraspecific interaction; (2) must be redundant, conspicuous, and stereotyped; and (3) may likely provoke an immediate response by the receiver benefiting the sender. From our behavioral data, we counted the number of behaviors executed. We identified visual displays of *Hylodes japi* based on reviews of anuran visual displays [2, 21]. The behaviors of three of the five new visual displays herein described (toes posture, head bobbing, and head snaking) were previously mentioned in literature [25, 33–35], but not included in the reviews of visual communication in anurans.

We recorded male advertisement and territorial calls with a Marantz digital recorder (PMD-660) coupled to a Sennheiser external unidirectional microphone (ME-66) positioned approximately 1.0 m from calling individuals. Courtship calls were obtained exclusively from video recordings. All calls were recorded with a sampling frequency rate of 44.1 kHz on 16-bit resolution. At the time of each recording, air and water temperatures were measured. We analyzed the data with the use of the software Raven Pro v1.4 (Cornell Lab of Ornithology, Bioacoustics Research Program). We described calls (except for the advertisement call which had previously been described [26]) by analyzing six parameters [36]: call duration (s), intercall interval (s), number of notes per call, note duration (s), internote interval (s), and dominant frequency range (kHz). We inferred the functions of each call according to the context in which they were emitted. We deposited recordings of advertisement, peep, and squeal calls in the Célio F. B. Haddad sound collection (CFBH; S1 Calls), located in Departamento de Zoologia, Instituto de Biociências of Universidade Estadual Paulista (UNESP), Rio Claro, São Paulo, Brazil.

We performed an analysis of limb and vocal sac use in signaling towards intruder males, particularly during short-range signaling. We recorded which side of the body (which limb and which vocal sac) was used in signaling by resident males within three different situations: with intruder male at the left side of the resident male, in front of the resident male, or at the right side of the resident male. For this analysis we excluded those signals that could not be produced with using only one side of the body, i.e., advertisement calls (always emitted with the use of both vocal sacs), body stationary visual displays (except throat display, which is included in our analysis), and non-stationary visual displays. Visual display categories are provided below.

## Results

### Natural history and communication

The communication of *Hylodes japi* is based on visual, acoustic, and tactile signals. These signals are more easily observed when densities of males are high, making intraspecific interactions more frequent. We observed males calling in all months except October. However, the breeding season occurs at the end of the rainy season (February–April), when we observed males calling in chorus and performing visual displays. During this period we observed intense male-male interactions and competition. We recorded pair formation, courtship, mating, and oviposition only between February and April as well. We recorded three courtship events: two during the day (1500 and 1700 h) and one at night, before sunrise (0400 h); the first ended with rejection by the female, whereas the other two resulted in oviposition (see [26]). *Hylodes japi* was found to exhibit three diurnal peaks of calling activity: in the beginning of the day, starting one hour before sunrise (between 0500 and 0800 h); in the middle of the day (between 1000 and 1300 h); and in the afternoon, until one hour before sunset (between 1500 and 1700 h). Males strongly decrease calling activities after sunset, however, during the breeding period at least a few individuals call sporadically all night long. Males adopt and defend as territories land areas on the margins of fast-flowing streams as well as emergent rocks, trunks, branches, and leaves located on the margins or in the middle of these streams, for use as calling and courtship sites, and for feeding activity (see [26]).

### Visual display repertoire

Males and females perform rich repertoires of visual displays during intraspecific communication. We observed 68 *H*. *japi* individuals and recorded 18 distinct visual displays associated with four behavioral contexts: advertisement, long-range agonistic, short-range agonistic, and courtship. The advertisement context involves nonaggressive behaviors when only one male is calling or when two neighboring males are interacting acoustically (calling in antiphony) without territory invasion. When a resident male identifies a conspecific intruder inside his territory he immediately changes his behavior, most likely in defense of his territory boundaries, which we refer to as the long-range agonistic context. The short-range agonistic context is observed when two males are closely interacting, even without physical contact, with a resident male defending his territory against an intruder male. Finally, we considered the courtship context to be from the moment that a male perceives a female inside his territory to the moment that the couple enters an underwater constructed chamber to deposit eggs.

The observed males (*N* = 65) performed 18 visual displays involving movements with limbs (toes, feet, hands, legs, and arms), body, vocal sacs, and head, besides stereotyped walking and jumping displays. Males perform visual displays during advertisement, agonistic, and courtship contexts. The observed females (*N* = 3) performed three distinct visual displays, recorded only during courtship events and using hands, arms, and body. Five behaviors (toes posture, two-armed impulse, head bobbing, head snaking, and truncated walking) did not fit into any previously described display category, so they are described herein as new visual displays for frogs. All visual displays performed by *H*. *japi* are detailed in Table 1 as well as their respective functions, inferred via comparisons among contexts in which each display was executed.

**Table 1:** Visual displays of *Hylodes japi* during intraspecific communication (modified from [2, 21]).

Limbs	
**1.**	Toe trembling (*N* = 87): wiggling, twitching, or vibrating the toes rapidly and with the foot and leg motionless. Toes may be moved independently, without an order, or in sequence in a wave-like pattern. Sometimes, only the fourth or fifth toes are moved. Whitish-silver dorsal surface of toe tips is exposed during the display. Toe trembling is performed by males with toes of right or left feet. It is usually performed during courtship behavior (*N* = 52) or when the resident male perceives a conspecific invading his territory (*N* = 20). However, it is also performed during male-male close interactions (*N* = 8) and during advertisement (*N* = 7). Toe trembling is a short and long-range visual display, with courtship and agonistic functions.
**2.**	Toe flagging (*N* = 47): lightly raising toes from the substrate, keeping them raised, slowly flagging them gently in up and down movements, keeping foot and leg motionless. Toes may be moved independently, without an order. Sometimes, only the fourth or fifth toes are moved. Whitish-silver dorsal surface of toe tips is exposed during the display. Toe flagging is performed by males with right, left, or both feet/toes simultaneously. It is usually performed during courtship (*N* = 27), advertisement (*N* = 9; only with toes of right or left foot) or when resident male perceives a conspecific invading his territory (*N* = 9; more frequently with toes of both feet together). It is rarely observed during male-male close interactions (*N* = 2). Toe flagging is a short and long-range visual display, with courtship, agonistic, and advertisement functions.
**3.**	**Toes posture** (*N* = 23; Fig 1A): holding the foot up for some seconds with a frontal exposure of the dorsal surfaces of feet and toes; or holding the foot up for some seconds with toes curved down, exposing dorsal surfaces of toes. Whitish-silver dorsal surface of toe tips is exposed during the display. Toes posture is performed by males with right or left foot independently, or with both feet simultaneously. It is usually performed when a resident male perceives a conspecific invading his territory (*N* = 12). However, it is also executed during advertisement (*N* = 8) and rarely during courtship (*N* = 3). Toes posture is a long and short-range visual display, with agonistic, advertisement, and courtship functions.
**4.**	Foot shaking or hand shaking (*N* = 36): rapidly moving one foot (*N* = 26) or one hand (*N* = 10) in an up and down motion, with the leg or arm motionless. It is a high-speed display. Foot shaking and hand shaking are performed by males with right or left foot or hand. Foot shaking is usually performed when a resident male perceives a conspecific entering his territory (*N* = 8) or during advertisement (*N* = 7), but is also observed during male-male (*N* = 4) or male-female (*N* = 7) close interactions. Foot shaking is a long and short-range visual display, with agonistic, advertisement, and courtship functions. Hand shaking is performed during courtship (*N* = 6) or male-male close interactions (*N* = 3), but is also observed when a resident male perceives a conspecific invading his territory (*N* = 1). Hand shaking is a short-range visual display, exclusively with courtship and agonistic functions.
**5.**	Leg stretching (*N* = 15): stretching a back leg and keeping it stretched on the ground for some seconds. Leg stretching is performed by males with right, left, or both legs. It is performed when a resident male perceives a conspecific invading his territory (*N* = 12). Rarely executed during male-male close interactions (*N* = 3). Leg stretching is a long and short-range agonistic visual display.
**6.**	Foot flagging (*N* = 43): slowly raising one leg, extending it out and back, lightly performing an arc in the air, and returning it back to the resting position. Whitish-silver dorsal surface of toe tips accentuates the display. Foot flagging is performed by males with right or left leg, sometimes with regular alternation. It is observed in all behavioral contexts (advertisement, *N* = 21; short-range agonistic, *N* = 10; long-range agonistic, *N* = 9; and courtship, *N* = 3). Foot flagging is a long and short-range visual display, with advertisement, agonistic, and courtship functions.
**7.**	Arm lifting (*N* = 88): rapidly moving one arm up and down at chest level. Arm lifting is a high-speed display. Arm lifting is performed by males in all behavioral contexts (courtship, *N* = 26; short-range agonistic, *N* = 13; long-range agonistic, *N* = 9; and advertisement, *N* = 6) and by females exclusively during courtship (*N* = 34). Males and females perform arm lifting with right or left arms. Arm lifting is the most common female visual display. Arm lifting is a long and short-range visual display, with courtship and agonistic functions.
**8.**	Arm waving (*N* = 37): rapidly lifting one arm and waving it up and down in an arc forward, passing it in front of the head. When crossing in front of the face, the brown arm and hand contrast with the bright whitish-silver area on ventral half of the lateral side of head, producing a flashing signal for the conspecific receiver, which also contrasts with the background. It is performed by males during agonistic contexts (short-range agonistic, *N* = 10; and long-range agonistic, *N* = 7), but was recorded once during advertisement. It is performed by females during courtship (*N* = 19). Males and females use both right and left arms to perform arm waving. Arm waving is the second most common female visual display. Arm waving is a short-range visual display, with agonistic and courtship functions.
**Body stationary**	
**9.**	Body lowering (*N* = 3): lowering the whole body to the ground, totally hiding the cream-colored ventral body. Body lowering is rarely performed by males during male-male close agonistic interactions. Body lowering is a short-range visual display.
**10.**	Upright posture (*N* = 16): raising only the anterior part of the body by extension of the arms, exposing the cream-colored throat and chest. Upright posture is performed by males, usually during agonistic contexts (long-range agonistic, *N* = 7; and short-range agonistic, *N* = 6); however, it is also executed during advertisement (*N* = 3). Upright posture is a long and short-range visual display, with agonistic function.
**11.**	**Two-armed impulse** (*N* = 6; Fig 1B): boosting the whole body forward by impulse via an up and down movement with both arms simultaneously, moving the frog forward and raising the anterior part of the body. Necessarily the two-armed impulse ends in an upright posture, however, upright posture is not necessarily preceded by two-armed impulse. Two-armed impulse is performed by males in agonistic contexts (long-range agonistic, *N* = 3; and short-range agonistic, *N* = 3), being a long and short-range agonistic visual display.
**12.**	**Head bobbing** (*N* = 9; Fig 1C): performing a single down or up jerky movement with the head without lifting hands or feet off the ground or moving the body. Essentially it is performed by males preceding calling. Head bobbing is rarely performed, but was recorded in all social-behavioral contexts studied (long-range agonistic, *N* = 4; short-range agonistic, *N* = 2; advertisement, *N* = 2; and courtship, *N* = 1). It is a long and short-range visual display, mostly with agonistic function.
**13.**	Throat display (*N* = 146): rapidly inflating and pulsating of vocal sac, without apparent sound production. It is performed once or repeated several times in quick succession. Bright, whitish double vocal sacs are clearly exposed during throat display, contrasting with the background and function as flash signals. Throat display is performed by males, which inflate both, or just the right, or just the left vocal sacs. Throat display is by far the most performed visual display in *Hylodes japi* (often being performed repeatedly, pulsating). It is usually performed during male-male agonistic close interactions (*N* = 89) or during the courtship (*N* = 40). However, it is also observed during advertisement (*N* = 9) and when a resident male perceives a conspecific invading his territory (*N* = 8). Throat display is essentially a short-range agonistic and courtship visual display.
**14.**	**Head snaking** (*N* = 1; Fig 1D): rapidly approaching a conspecific female, raising the head up and moving it to alternate sides eight times (four times on each side), in a snakelike motion. It is performed with the throat at the level and in front of the female snout, with the two being very close to each other, but without touching. During head snaking sequential and conspicuous movements, the cream-colored throat and chest is closely exhibited to the female. Head snaking was recorded once, when performed by a male during courtship behavior. It is displayed preceding underwater courtship behaviors, when male is leading the female. Despite being very rare and difficult to observe, head snaking must occur commonly during courtship; moreover, another report of the same behavior for *Hylodes phyllodes* (see discussions) provides additional evidence. Head snaking is a very characteristic behavior and fits conditions of being a visual display. Head snaking is a short-range courtship visual display.
**15.**	Body raising (*N* = 3): raising the body by extension of legs and arms. Body raising is a posture performed by males. Rarely performed, it was recorded during male-male close interactions (*N* = 2) and advertisement (*N* = 1). Body raising is a short and long-range visual display, with agonistic functions.
**16.**	Body jerking (*N* = 51): performing a jerky movement with the body, without lifting either hands or feet. It is performed in a forward and backward or up and down motion. Body jerking is usually performed by males in agonistic contexts (long-range agonistic, *N* = 16; and short-range agonistic, *N* = 14); however, it is observed during courtship (*N* = 11) and was registered once during advertisement. It is also performed by females in courtship (*N* = 9). Body jerking is a short and long-range agonistic and courtship visual display.
**Body non-stationary**	
**17.**	**Truncated walking** (*N* = 13; Fig 1E): lowering the body and walking ahead slowly, with alternation of legs and arms. Truncated walking is performed with a moving and stopping pattern. The right arm is moved concomitantly with the left leg and vice-versa. Truncated walking is performed by a resident male when he perceives a conspecific invading his territory. Most likely it is performed when a resident male is approaching the intruder. Truncated walking is a long-range agonistic visual display.
**18.**	Jump display (*N* = 3): jumping quickly and sideways in front of a conspecific male, as a discontinuous movement. It is performed with one or more conspicuous quick jumps, none of them to the same side. Jump display is rarely performed, being recorded only three times by males during short-range agonistic interactions. Jump display is a short-range agonistic visual display.

All displays were recorded in advertisement, agonistic, and courtship contexts (see text for definitions), and the number of observations for each display is indicated by *N*. New visual displays for frogs are highlighted in bold.

**Fig 1:**
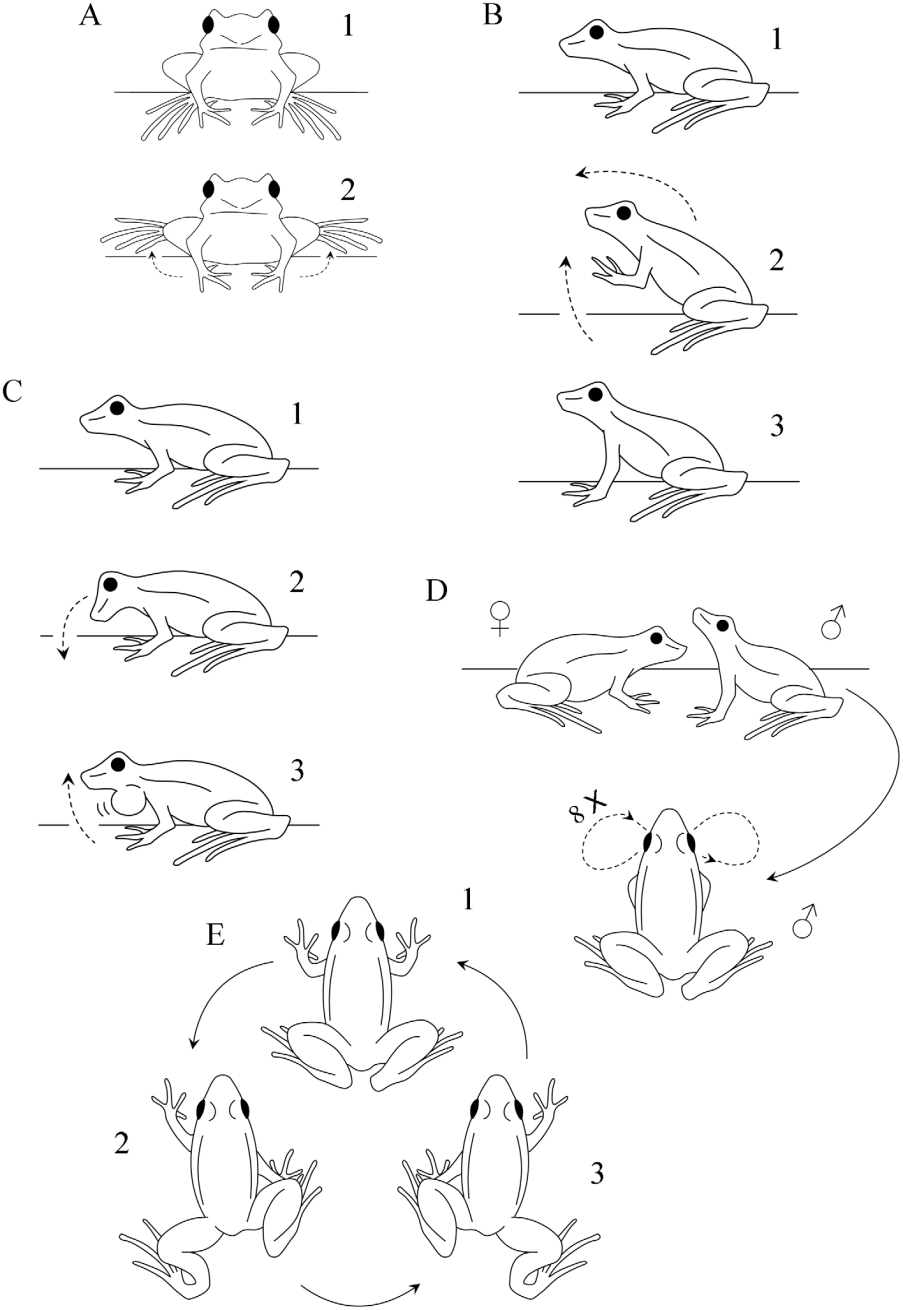
New visual displays performed by male *Hylodes japi*. (A) Toes posture; from resting position (above; frontal view) raising feet and holding feet up for some seconds, exposing dorsal surfaces of toes (below; frontal view). (B) Two-armed impulse; from resting position (above; lateral view) boosting the whole body forward by impulsion via an up and down movement with both arms simultaneously, moving the body forward and raising the anterior part of the body. (C) Head bobbing; from resting position (above; lateral view) performing a single down or up jerky movement with the head without lifting either hands or feet off the ground nor moving the body; it is performed preceding calls by males. (D) Head snaking; rapidly approaching a conspecific female, raising the head up (above; lateral view of the couple) moving it to alternate sides eight times (four times each side), in a snakelike motion (below; dorsal view of the male); it is performed with the throat at the level and in front of the female snout, with the frogs being very close to each other, but without touching. (E) Truncated walking (dorsal view); lowering the body and walking ahead slowly, with alternation of legs and arms; it is performed with a moving and stopping pattern; left arm is moved concomitantly with right leg and vice-versa.

### Acoustic signal repertoire

**Fig 2:**
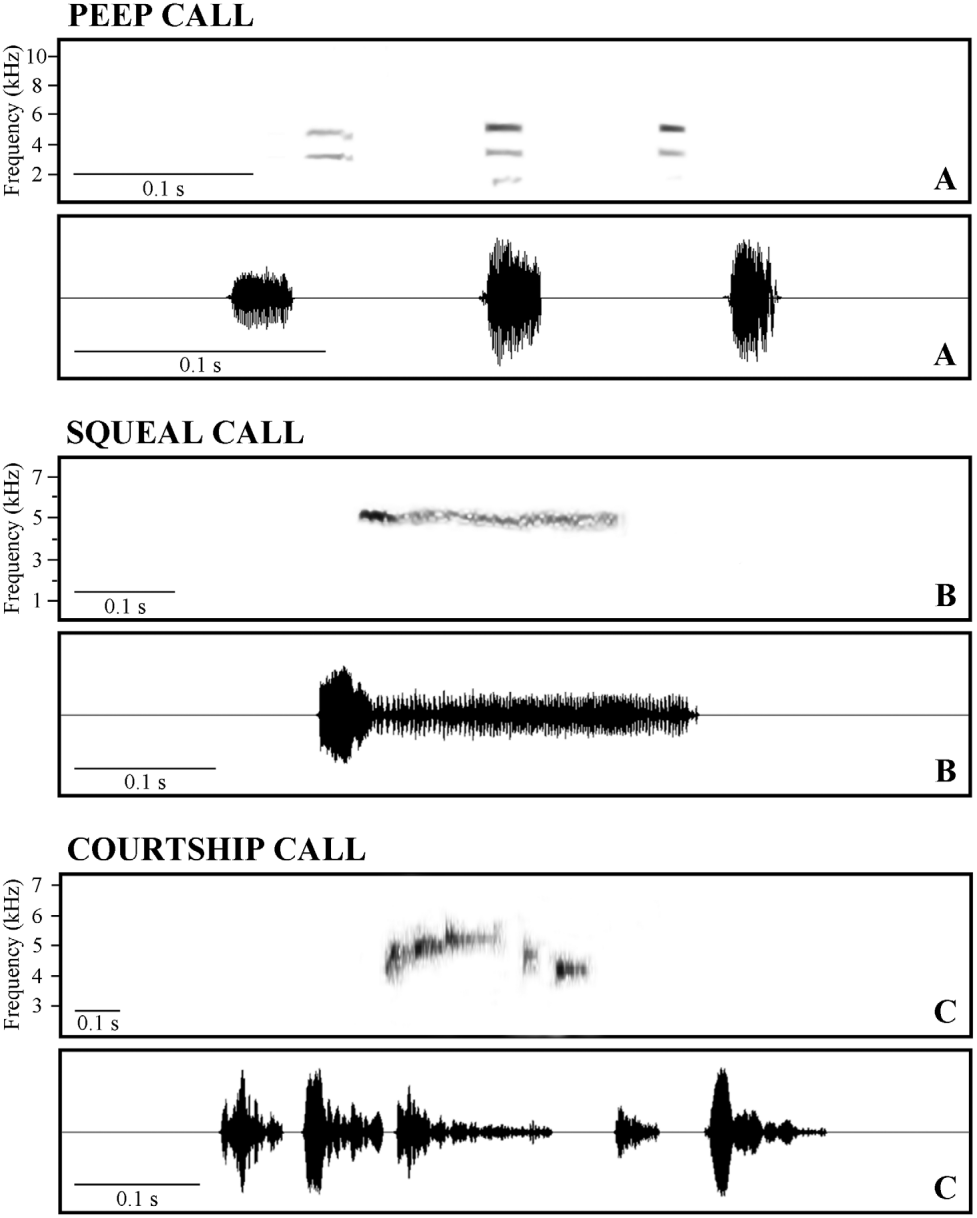
Calls of male *Hylodes japi*. (A) Spectrogram (top) and waveform (bottom) of a peep call composed of three notes. (B) Spectrogram (top) and waveform (bottom) of a squeal call. (C) Spectrogram (top) and waveform (bottom) of a courtship call composed of five notes. Peep and squeal calls recorded on 15 April 2011 and courtship call on 6 March 2012. For all calls, air temperature was 24°C and water temperature 19.5°C.

*Hylodes japi* also exhibits a diverse acoustic repertoire that, besides the advertisement call (see [26]), is composed of three other male call types: peep (Fig 2A), squeal (Fig 2B), and courtship calls (Fig 2C). The peep and squeal are territorial calls and both have a courtship function. The courtship call is triggered by tactile or visual-tactile stimulation by the female (*N* = 15) during courtship (see below). Parameters of each call type at an air temperature of 24°C and a water temperature of 19.5°C, are described below.

Peep calls are composed of frequency modulated peep notes. Peep call duration is 0.03–0.38 s (0.21 ± 0.072, *N* = 34 calls from five males). Calls are emitted at intervals of 1.04–1.86 s (1.37 ± 0.23, *N* = 31 intervals from four males). Each call is composed of 1–4 notes (2.63 ± 0.71, *N* = 32 calls from five males). Note duration is 0.019–0.042 s (0.028 ± 0.005, *N* = 90 notes from 36 calls from five males). Notes given at intervals of 0.05–0.11 s (0.075 ± 0.014, *N* = 90 intervals of 45 calls from five males). The dominant frequency occurs in the third harmonic and ranges from 3.1–5.5 kHz (4.9 ± 0.54, *N* = 90 call notes from five males).

Peep and squeal calls can be emitted in combination, with a peep preceding a squeal call. The duration of squeal calls is 0.037–0.33 s (0.17 ± 0.086, *N* = 30 calls from five males). Squeals are emitted at intervals of 0.6–7.46 s (1.23 ± 1.23, *N* = 30 intervals, between the end of a squeal call and the beginning of a peep call, from five males). They are composed of a single note (*N* = 30 calls from five males). Notes have the dominant frequency ranging from 3.1–5.5 kHz (4.8 ± 0.58, *N* = 30 call notes from five males).

Duration of courtship calls is 0.24–0.88 s (0.46 ± 0.17, *N* = 11 calls from one male). Each call has 4–6 notes (5.28 ± 0.65, *N* = 11 calls from one male). Note duration is 0.016–0.098 s (0.055 ± 0.02, *N* = 55 notes from 11 calls from one male). Notes given at intervals of 0.012–0.19 s (0.037 ± 0.041, *N* = 43 intervals of 11 calls from one male). Each call is composed of frequency modulated notes, usually rising until the middle of the call and then lowering at the end. Dominant frequency is in the third harmonic and ranges from 3.6–5.7 kHz (4.9 ± 0.51, *N* = 57 call notes from one male).

### Male communication: Territoriality and signal choice

Males defend territories as breeding sites (calling and courtship). When a male perceives the presence of a conspecific male or female inside his territory, he readily increases emission of peep and squeal calls, while also intensifying the production of visual displays (e.g., S2 and S3 Movies).

**Fig 3:**
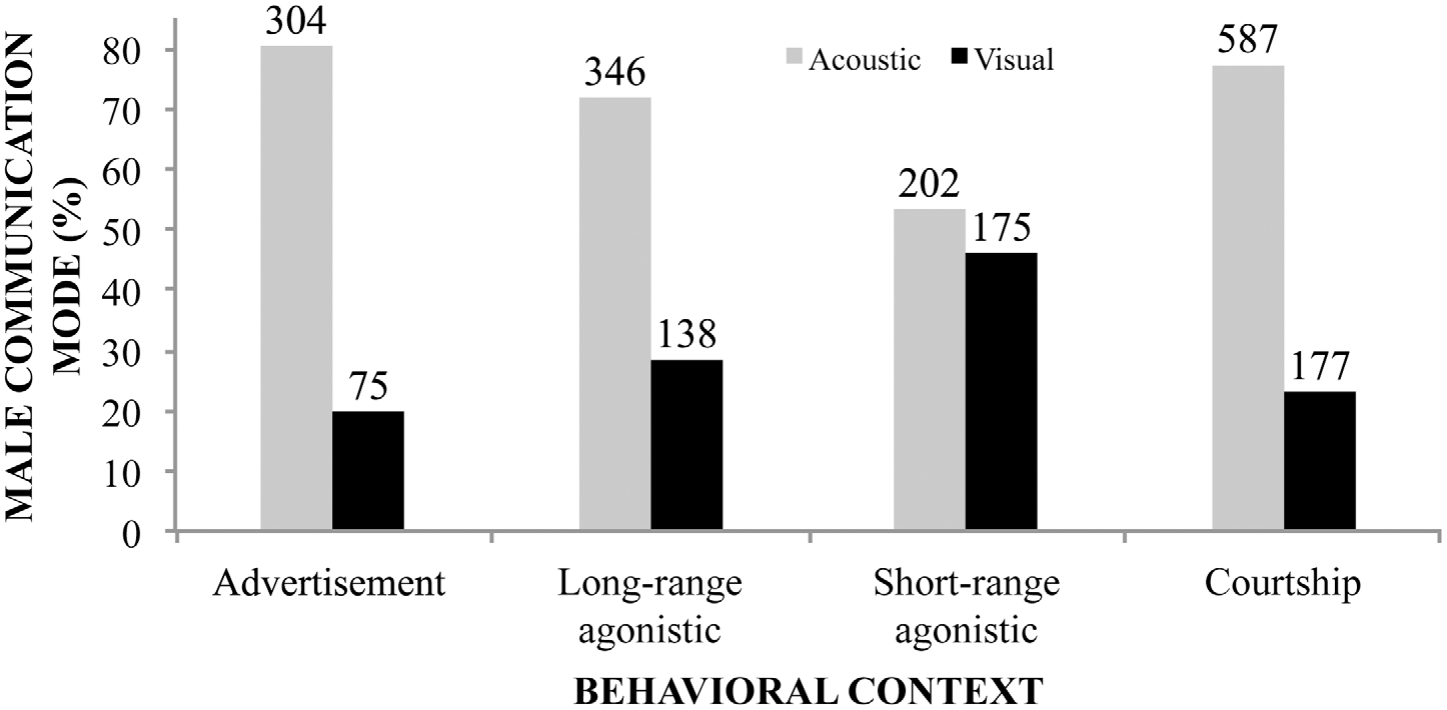
Frequency of visual and acoustic signals performed by *Hylodes japi* males in distinct contexts. We observed males in advertisement (*N* = 28 males observed), long-range agonistic (*N* = 10 males), short-range agonistic (*N* = 24 males), and courtship contexts (*N* = 3 males). Values on top of each bar are the number of observations.

**Fig 4:**
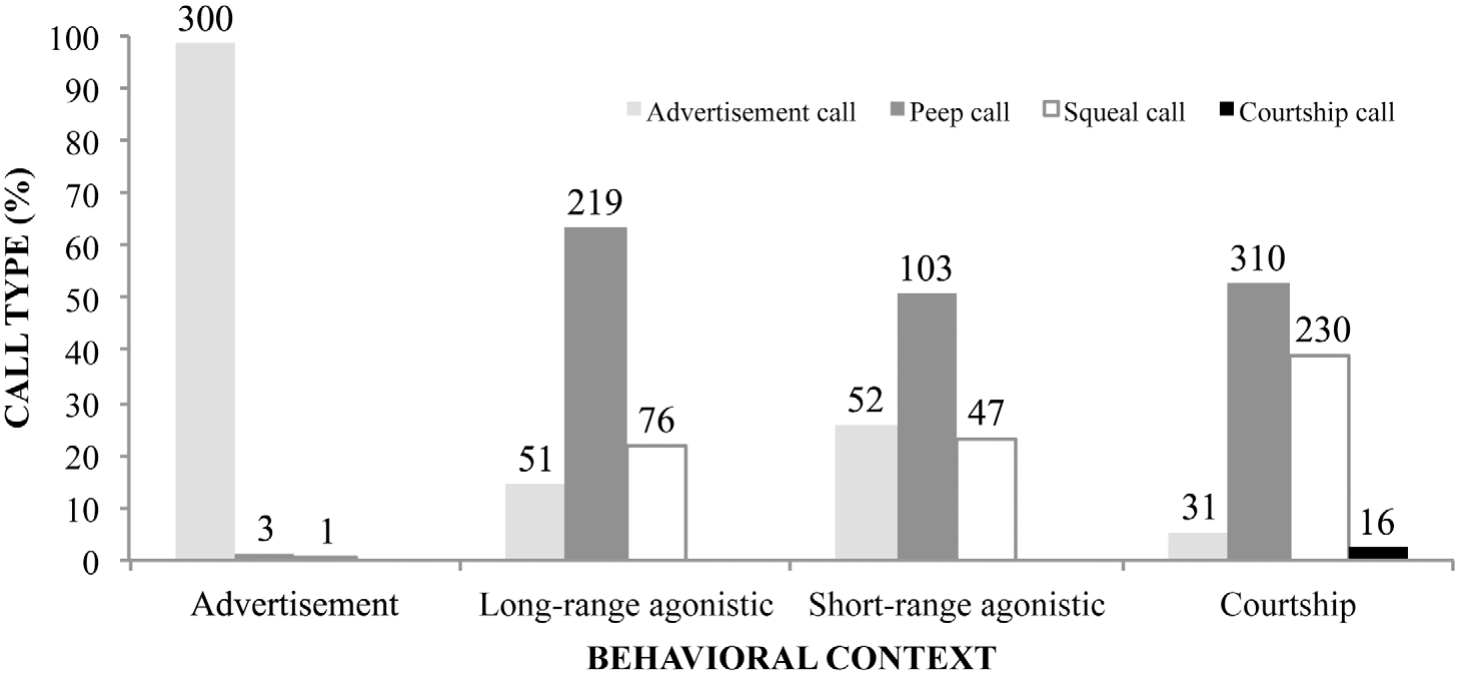
Frequency of advertisement, peep, squeal, and courtship calls of *Hylodes japi* in distinct contexts. We observed males in advertisement (*N* = 28 males observed), long-range agonistic (*N* = 10 males), short-range agonistic (*N* = 24 males), and courtship contexts (*N* = 3 males). Values on top of each bar are the number of observations.

Of the 65 males observed, 28 were engaged in advertisement context, 10 in long-range agonistic, 24 in short-range agonistic, and three in courtship. Males exhibited a higher proportion of acoustic signals than visual displays in all behavioral contexts, except for short-range agonistic, in which visual and acoustic signals were almost equally common (Fig 3). We also found differences in call types emitted according to each social context (Fig 4). During advertisement, males basically emit advertisement calls by inflating both vocal sacs. The advertisement call is the least emitted acoustic signal in long-range agonistic contexts, but has almost the same proportion as squeal call in short-range agonistic contexts. In long-range and short-range agonistic contexts, the peep call is the vocalization most frequently emitted. Although only emitted during courtship, the courtship call is the least frequent call type within that context. During male-female close interactions, peep and squeal calls are most frequent.

There are also differences among the four distinct contexts in the visual displays used by males (Table 2), as shown by the following examples. Foot flagging is a visual advertisement display, but it was also recorded in the other three contexts. Arm waving is performed by males with exclusive agonistic function and by females with exclusive courtship function. Throat display is a significant visual display for short-range agonistic and courtship behaviors, despite being executed in advertisement and long-range agonistic contexts (see S4 Movie). Head snaking is used by males only for a specific moment during courtship; when the male is leading the female to dive into the water.

**Table 2:** Percentage of visual displays performed by *Hylodes japi* males in each behavioral context.

Visual display	Behavioral context			
	Advertisement (%)	Long-range agonistic (%)	Short-range agonistic (%)	Courtship (%)
**Toe trembling**	9.3	14.5	4.6	29.4
**Toe flagging**	12	6.5	1.1	15.3
**Toes posture**	10.7	8.7	0	1.7
**Limb shaking**	9.3	6.5	4	7.3
**Leg stretching**	0	8.7	1.7	0
**Foot flagging**	28	6.5	5.7	1.7
**Arm lifting**	8	6.5	7.4	14.7
**Arm waving**	1.3	5.1	5.7	0
**Body lowering**	0	0	1.7	0
**Upright posture**	4	5.1	3.4	0
**Two-armed impulse**	0	2.2	1.7	0
**Head bobbing**	2.7	2.9	1.1	0.6
**Throat display**	12	5.8	50.9	22.6
**Head snaking**	0	0	0	0.6
**Body raising**	1.3	0	1.1	0
**Body jerking**	1.3	11.6	8	6.2
**Truncated walking**	0	9.4	0	0
**Jump display**	0	0	1.7	0

We observed males in advertisement (*N* = 28 males observed), long-range agonistic (*N* = 10 males), short-range agonistic (*N* = 24 males), and courtship behavioral contexts (*N* = 3 males).

*Hylodes japi* males use acoustic and visual signals to maintain territories, thereby avoiding fights. Most likely, fights happen when visual and acoustic signals do not work in deterring territorial invasions. We observed a physical confrontation between two males, apparently disputing the same territory. In March 24 2011, two *H*. *japi* males were on separate small emergent rocks in the middle of a fast-flowing stream. The frogs were close to each other (50 cm apart) and using aggressive visual and acoustic signals, when one of them jumped over to the rock of the other. The individual that jumped was clearly larger (males were not measured) and pushed the smaller individual with his snout and chest using forward and backward movements, during which both males were emitting squeal calls. The smaller male apparently tried to stay on the rock, however, it was pushed off and into the water. That pushing fight lasted about 50 min. The smaller male tried to climb up onto the rock again, simultaneously pushing his opponent using his snout and body. They kept in that dispute for 10 more min until the larger male again pushed the smaller male back into the water, thereby winning the contest.

### Female-male communication: An elaborate courtship with bimodal signaling

During our study we observed three couples in courtship for a total of 53 min. Although these courtship events were only partially observed, put together they provide the entire sequence of behaviors. *Hylodes japi* courtship is complex and comprises visual, acoustic, and tactile signals (see S5 Movie). Males call throughout the courtship, alternating among advertisement, peep, squeal, and courtship calls. During courtship, we observed males performing visual displays using toes, feet, hands, legs, arms, vocal sacs, head, and body. Females also performed visual displays, however, only with the movements of hands, arms, and body.

Courtship takes place on land and always on the margin of a fast-flowing stream or close to it inside the male’s territory, except for the part that precedes oviposition, which occurs in a chamber constructed under water on the bottom of the fast-flowing stream. The complete courtship of *H*. *japi* may be summarized into three steps as follows: (1) evaluation, (2) acceptance, and (3) leading. These steps are described in detail below.

**Fig 5:**
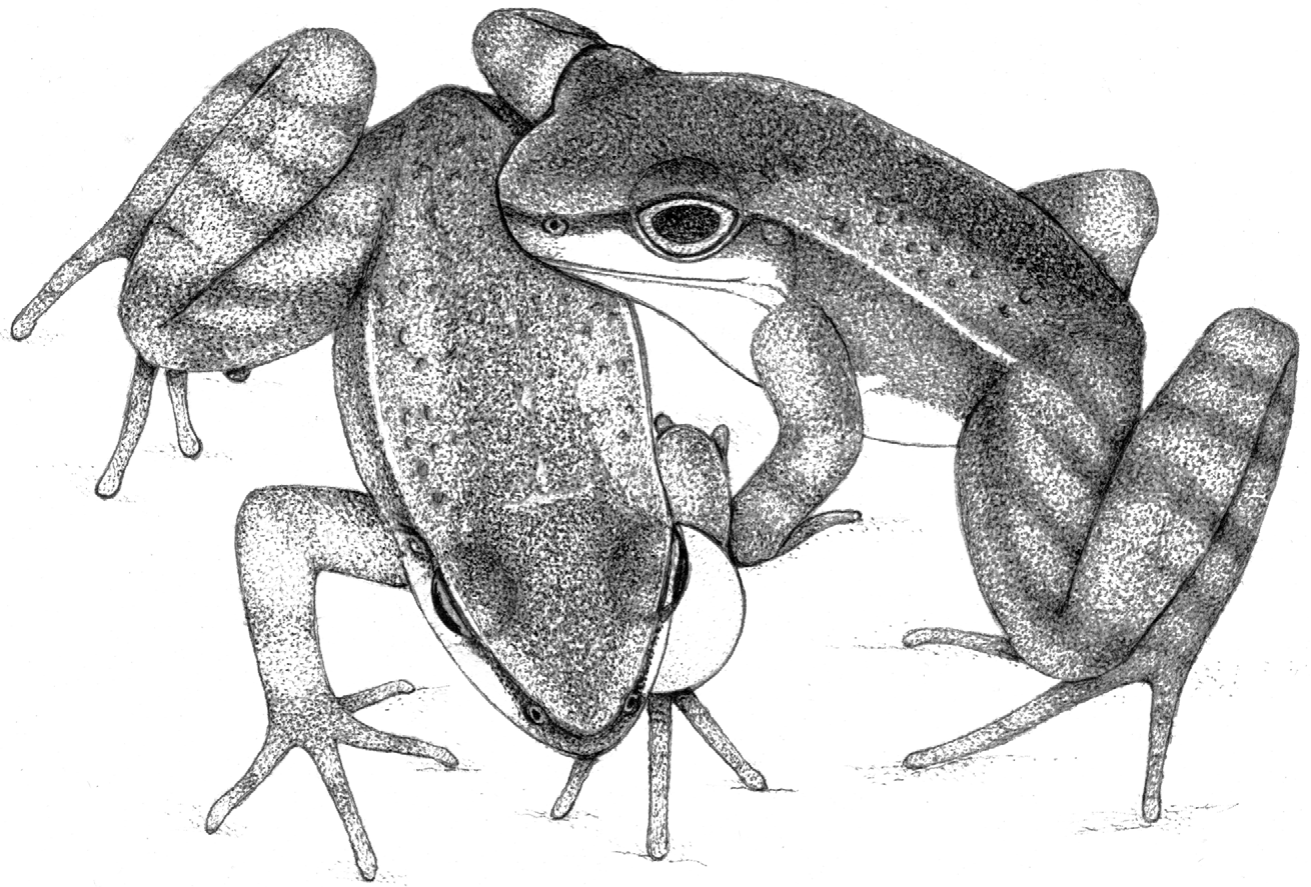
Mating couple of *Hylodes japi* during courtship. *Hylodes japi* female touching the dorsum of the conspecific male with her gular region during courtship (drawn based on images captured by video recording). Male is calling only with one vocal sac inflated, the one closest to the female (left vocal sac), also showing the visual component of his bright whitish vocal sacs. Note the female’s left arm is closer to the male’s head and the female’s right arm is closer to his posterior body region.

(1) Evaluation: the female was attracted by the advertisement calls of a male and entered his territory and observed him from afar. Immediately after perceiving the presence of the female, the male stopped emitting advertisement calls, started to perform visual displays, and drastically increased emission of peep and squeal calls (see Fig 4). At this point, the male emitted advertisement calls only if a conspecific male approached the mating couple. The female slowly started to approach the male by sporadic jumps. Sometimes the male also jumped towards the female. During the approaching process, which lasted about two min, the male faced the female and alternated between acoustic and visual communication modes. The male started courtship by performing visual displays (foot shaking and throat display). Then he alternated between peep call, squeal call, and visual displays, including toe trembling, toe flagging, toes posture, foot shaking, hand shaking, and foot flagging. As a result of female evaluation, there were two mutually exclusive possibilities: the female refused that male and left his territory (*N* = 1); or female accepted that male and approached him even more (*N* = 2). When a male was refused, the female dived into the fast-flowing stream and stayed motionless on the bottom. The male started to pursue the female and dived into the stream and approached her. The female, however, left by emerging and diving, yet still followed by the male, until she completely fled, leaving the area by swimming into the benthic zone of the fast-flowing stream. Subsequently, the male emerged and returned to advertisement context (pre-courtship), emitting advertisement calls and most likely waiting for another potential mate.
(2) Acceptance: when a female was interested in a male, she stayed at his side. At that moment, the female started signaling by arm lifting. This visual display ended with a tactile signal, when the female putted one of her arms down over one of the male’s feet, thereby touching him. Immediately after that the female touched the male’s dorsum (next to his head) with her gular region. The female intermittently putted her hands on the male’s dorsum, but kept her gular region continuously over the male until the next step of courtship (leading) (see Fig 5 and S5 Movie). The male continued to perform intensively both peep and squeal calls (usually with regular alternation), intercalating them with visual displays (toe trembling, toe flagging, foot shaking, hand shaking, arm lifting, head bobbing, throat display, and body jerking), while the female remained motionless. Without an apparent order, the female performed visual-tactile signals: arm lifting, or arm waving (both usually ending with female touching the face of the male or his anterior or posterior dorsal body region, depending on which arm the female uses to perform the signal; Fig 5 and S5 Movie show the position of the couple), or body jerking. Even while performing visual-tactile signals, the female kept her gular region on the male’s dorsum. When stimulated by female visual-tactile signals, oftentimes the male readily responded with courtship calls (*N* = 15; see S5 Movie). Male and female continued with the visual-acoustic-tactile stimulation process for around 40 min. Once during the courtship behavior step, the male performed body jerking and the female responded by slightly moving her body forward and backward few times, consequently scrubbing her gular region on the male’s back. As the acceptance step nears an end, the female continued to keep, simultaneously, her gular region and both hands on the male’s back.

**Fig 6:**
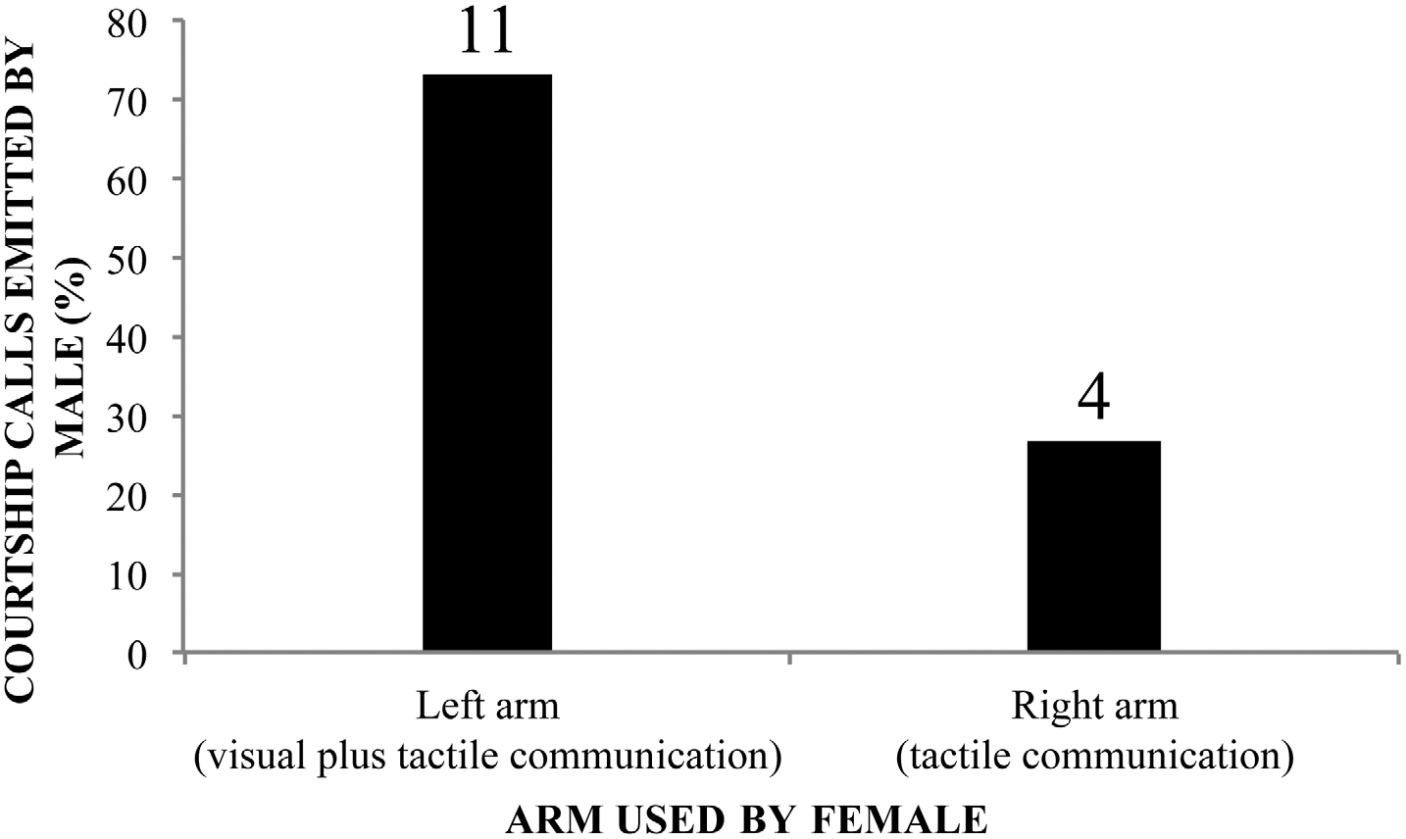
Male courtship calls triggered by distinct female stimulus. Percentages of courtship calls emitted by the male when the female performs bimodal signals (visual plus tactile communication) and pure tactile signals, in *Hylodes japi*. See text for details. Values on top of each bar are the number of observations.

Courtship calls are emitted only during the acceptance step of courtship. From a video recording of one of the three courtship events observed, we quantified signals displayed by females and which arms where used to perform arm lifting or arm waving (analyzed together). With this couple, we observed that the female’s left arm is closer to the male’s head and the female’s right arm is closer to the posterior body region of the male (see positions of male and female during the acceptance step in Fig 5 and S5 Movie). The male is able to see only those movements performed by the female’s left arm. So, movements performed by the female’s right arm could only be perceived by the male through tactile stimuli. Fig 6 exhibits frequencies of courtship calls emitted by the male (*N* = 15), triggered by movements of the left and right arms of the female. We observed that both of the female’s arms stimulated the male to emit courtship calls, however, the left arm stimulated 11 whereas the right arm only four.

(3) Leading: there were two possible scenarios in this courtship step; male and female already were located on margin of the fast-flowing stream (*N* = 2) or they were near the margin (*N* = 1). In the first case, maintaining physical contact with the female (the female keeping her gular region on the male’s back), the male slightly moved his body forward, consequently moving the female’s body as well. Immediately, the male dived into the stream followed by the female (see S5 Movie). In the second case, the male leaded the female to the place where they dived. In this case, the male jumped in the direction of the stream, attracting the female by using emissions of advertisement, peep, and squeal calls (in similar proportions). When the male and female were about 2 cm apart, they kept facing each other for some seconds. Then, the male jumped right in front of the female, raised his head, performed head snaking display, and jumped on the stream margin, now moving to about 30 cm away from the female. The male turned to the stream and, emitting only advertisement calls, attracted the female, who slowly approached the male by sporadic jumps until about 1 cm from him. When female arrived on the margin, the male slowly entered in the water and dived next to the margin, and the female followed. They examined the underwater area close to the male’s territory and made a decision on where to construct the chamber, which the male began to excavate (see [26]). From this moment on we did not detect any more visual signaling. The underwater part of the leading step was composed of tactile stimulation of mutual touches between the couple (described more detailed in [26]). The leading step lasted around 11 min; six min on land and five min in the water until the couple entered in the chamber. Inside the chamber, oviposition lasted 27 min. After oviposition, concealing of the chamber by the couple lasted five min. From the moment when the male first perceived the female inside his territory to the moment that the chamber is filled with eggs and sealed, courtship took 85 min, with 48 min on land and 37 min underwater.

### Male communication: Signal emission control and directionality

Males of *Hylodes japi* move several times while calling during advertisement contexts, usually at an angle of 45°, most likely making themselves more detectable to both male and female. Furthermore, we observed two situations where males were calling partially submerged in the stream so that their inflated vocal sacs were in contact with water surface during emission of advertisement calls. The inflated vocal sacs touching the water surface produce distorted calls (*N* = 2). In one of those situations, the male apparently perceived the distorted sound that was being produced and, during the intervals between his advertisement calls, experimented with new calling positions, and tried to get out of the water, apparently looking for a better position to call until producing the regular call without the contact of his vocal sacs with the water surface (S1 Movie). We also audibly observed that the males are able to control the intensity of their calls, sometimes clearly lowering vocalization volume during short-range agonistic interactions or courtship interactions.

Finally, visual displays performed by limbs are infrequently executed with both arms or legs at the same time (*N* = 20 displays for both limbs simultaneously, from 376 displays observed for 65 males); it seems that individuals choose one of the sides (right or left) to perform the display. In addition, males apparently have control over which vocal sac they will use during acoustic and visual signaling. When calling or performing the throat display, the male chooses to use both vocal sacs simultaneously or only one of them individually. However, the advertisement calls of *H*. *japi* are always executed by using both the vocal sacs (*N* = 329 advertisement calls video recorded, from 65 males). From video recording analysis of 65 males, we also observed that throat display and the other calls in the repertoire (peep, squeal, and courtship) can be executed with both vocal sacs simultaneously (*N* = 18 throat displays, 314 peep calls, 124 squeal calls, and 6 courtship calls), only with the right vocal sac (*N* = 14 throat displays, 30 peep calls, 7 squeal calls, and none courtship calls), or left vocal sac (*N* = 72 throat displays, 190 peep calls, 174 squeal calls, and 10 courtship calls).

**Fig 7:**
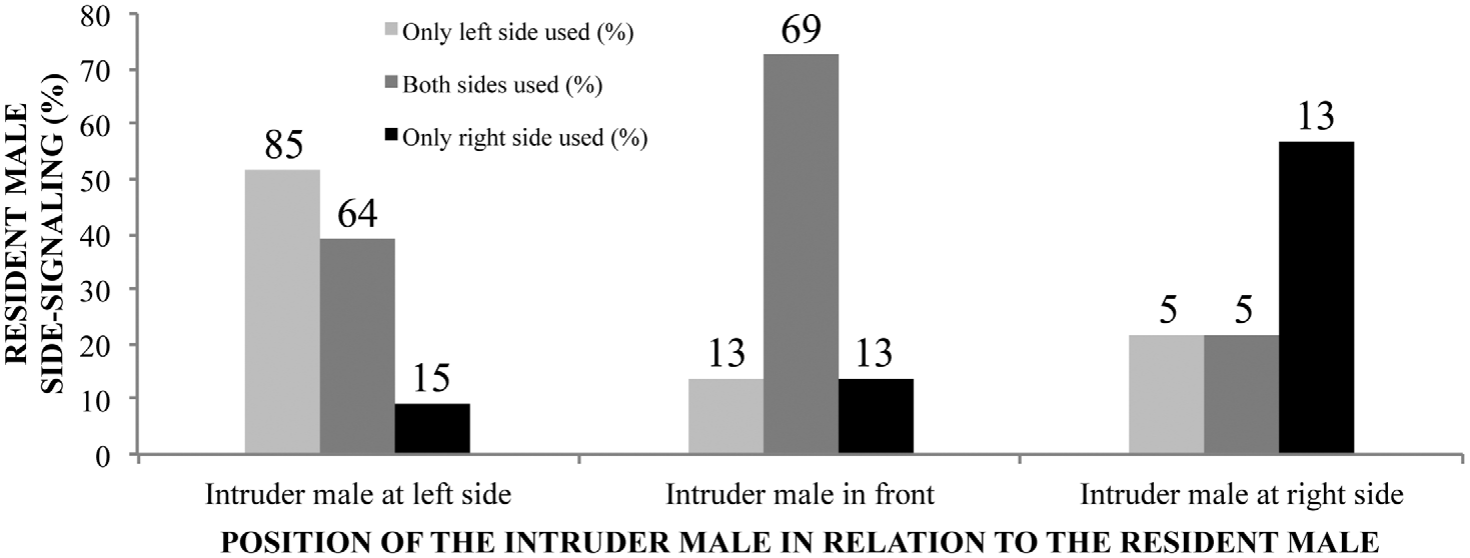
Resident male of *Hylodes japi* chooses left or right limb/vocal sac to visually signal. In the short-range agonistic context, we recorded behaviors from resident males (*N* = 24) in three distinct situations. We observed behaviors when an intruder male is at the left side of the resident male (*N* = 9 males and 164 communication signals recorded), in front of the resident male (*N* = 10 males and 95 communication signals recorded), and at the right side of the resident male (*N* = 5 males and 23 communication signals recorded). Values on top of each bar are the number of observations.

We conduct an analysis of limb and vocal sac usage in short-range signaling to intruder males (see Materials and Methods) and provide our results in Fig 7. When resident males decide how to emit calls or perform displays that can be signaled by both, left, or right vocal sacs or limbs, they do it based on conspecific receptor position, during close-agonistic interactions (see S4 Movie).

## Discussion

*Hylodes japi* exhibits sophisticated intraspecific communication involving a rich repertoire of visual displays and acoustic signals, which is even more complex during courtship when individuals also include tactile signaling.

*Hylodes japi* is cryptic, with brown and reddish brown dorsal colorations, which resemble the background and substrates where it lives. However, when observed from frontal view (as they are observed by conspecific individuals), their cream-colored venter contrasts with the dark background. Similar dorsal and ventral body color patterns are found in other members of the family Hylodidae (e.g., see [6, 21, 37, 38]). While dorsal coloration works as camouflage for predators, a contrasting ventral pattern can serve to convey intraspecific visual cues, in particular for visual displays such as body lowering, upright posture, and head snaking. These three displays are associated with, respectively, subordination, territoriality, and female stimulation, suggesting that ventral body color may convey visual messages during intraspecific interactions. During body lowering, a male hides his cream-colored venter, which can be interpreted as a submissive display, consequently avoiding agonistic behaviors and fights by expressing non-aggressive intentions during short-range territorial interactions with conspecific males [2]. Upright posture and head snaking are visual displays that convey, respectively, aggressive and courtship messages via the exposure of a venter with contrasting coloration.

Movements and postures, alone or in combination, are additional conspicuous traits that can shape visual displays [2, 7]. Head snaking is a stereotypic movement intensified by the cream ventral coloration. Foot and hand shaking, arm lifting, head bobbing, body jerking, and jump display are examples of visual displays that are conspicuous due to movements. Leg stretching, two-armed impulse, and truncated walking are conspicuous due to the association of movements and postures. Body raising is a conspicuous display due to posture. Toe trembling, toe flagging, and foot flagging are composed of movements that are made more evident by the contrasting bright whitish-silver color of toe tips. In toes posture, the posture adopted by the male also exposes the contrasting color of toe tips.

Arm waving, performed by *H*. *japi* females and males, is a visual display involving movement and color association. In *Hylodes*, arm waving is performed with a quick movement, producing a flashing signal by passing the brown arm in front of the bright whitish-silver area on ventral half of lateral side of head (S2 Movie). Likewise, movement and color associations for visual signaling have been observed in other frog genera. *Brachycephalus ephippium* (family Brachycephalidae) and *Atelopus zeteki* (family Bufonidae) are diurnal frogs that also perform arm waving, however, in these cases the display is somewhat different, being performed slowly by passing yellow arms in front of their black eyes [39, 40]. Variations of the same visual display (including variations in the combinations of colors, movements, and postures) can be identified, and are more apparent among different phylogenetic groups of frog.

Throat display is another case of visual signaling involving movement and color association in *H*. *japi*. Throat display is described as movements of the bright, whitish vocal sacs, which also produces flashing signals. It has been demonstrated that frogs are voluntarily able to control their vocal sac (e.g., [41]). Here we add that *H*. *japi* males are able to direct their double vocal sacs individually toward the conspecific receiver, which is a novelty among frogs. Likewise, it is known that frogs are voluntarily able to choose which limb will be used for signaling (e.g., [6, 23, 37]). Recently, male *Micrixalus kottigeharensis* (previously *Micrixalus saxicola*) were found to perform foot-flagging, directing the displays toward the interacting male [42]. In addition to vocal sac control and orientation, our findings suggest that *H*. *japi* males, while visually displaying, also make decisions on which limb will be used according to the position of the conspecific receiver. Furthermore, the control and direction of signal emission by males are evidenced by the 45° turns that they make during advertisement contexts, call volume control during short-range intraspecific interactions, better positioning when advertisement call is distorted (S1 Movie), and selection of the communication mode or signal type that better fits in a particular behavioral context. When a male is controlling and directing his signals, there is most likely a trade-off between energy demands and success in transferring information. It is likely that signal control improves and optimizes individual performance of males. Potentially, similar results can be expected for other visually signaling frogs from different continents.

We observed that vocal sac control is executed either when performing a visual display (throat display) or when emitting acoustic signals (peep, squeal, and courtship calls), with the exception of the advertisement calls (which are always emitted with use of both vocal sacs; S2 Movie). Most likely there were not any evolutionary pressures for the development of vocal sac control (to direct visual signals) associated with advertisement call. This particular call type is usually emitted in advertisement contexts, when a male is alone within his territory (i.e. there are not receivers around the signaling male; e.g., see [43]). Another possibility is that, most likely, advertisement calls need to be produced at higher intensities and both vocal sacs are used to maximize the radiating surface of the frog. During advertisement call emission (usually a long-range signal with the dual functions of territorial maintenance and female attraction), the acoustic component may be more important than the visual component. The signal is purely acoustic if the receptor cannot see the emitter; from the moment that the receptor sees the emitter the signal becomes bimodal, with the addition of visual information to the acoustical information (by the pulsating of vocal sacs; e.g., see [19, 44–46]). In particular during close intraspecific interactions, a male may use his vision and/or hearing to locate a conspecific receptor and direct his vocal sacs during signal emission. The bright vocal sacs may act as visual cues either when males are performing the pure visual display (throat display, a unimodal visual communication) or when males are calling. During calling, color and movement of the vocal sacs (visual components) and the call sound (acoustic component) may act concomitantly as a fixed composite signal (sensu [47]) in bimodal communication. It is reasonable to suppose that color and movement of the vocal sacs are not only an epiphenomenon of the acoustic signaling, but they do help with detection and discrimination of the emitter within the complex background noise of the habitat [45, 48].

We report two visual displays performed by the female during the courtship behavior that are usually integrated with tactile signaling: arm lifting and arm waving. Tactile and visual components of the movements performed by the female act together in synergy, working as a bimodal signal. This is the second evidence of bimodal communication in *H*. *japi* (the first is discussed in the preceding paragraph). As with visual-acoustic communication, if the male (receptor) cannot see the visual component, the female signal is purely tactile; if the male sees her movement during the tactile-visual signaling, the signal becomes bimodal. The tactile component of the female signal is by itself enough to trigger the male courtship call. However, when tactile and visual components are combined as a unique signal, they are more likely to succeed in male stimulation, with three-times more positive male replies. We conclude that movement is not only an epiphenomenon of tactile signaling, but rather a visual component that amplifies the tactile component during female-male communication, with an increase in the accuracy of the transferred message [47, 49, 50]. Our study is the first to describe the combination of visual and tactile signals in the courtship behavior of frogs.

By comparing our data with that in the literature, we recognize similarities in communication among species in the genus *Hylodes*. Repertoire and functions of acoustic signals are alike among *H*. *japi* (present study), *H*. *asper* [6], *H*. *heyeri* [51], *H*. *meridionalis* [52], and *H*. *phyllodes* [53]. Tactile stimuli executed by males and females are usually observed during courtship in other *Hylodes* species as well (e.g., *H*. *asper* and *H*. *phyllodes*; [6] and [34], respectively). Current data concerning intraspecific communication suggests that there is a general behavioral pattern in the genus *Hylodes*. Moreover, these three communication modes (visual, acoustic, and tactile) are also recorded as courtship behaviors of other frog families in which the male leads the female to an oviposition site. Some examples are: *Allobates femoralis* (Aromobatidae; [54]), *Ameerega braccata* (Dendrobatidae; [55]), *Aplastodiscus arildae* (Hylidae; [8]), *Aplastodiscus leucopygius* (Hylidae; [56]), *Aplastodiscus perviridis* (Hylidae; [57]), *Leptodactylus fuscus* (Leptodactylidae; [58]), and *Leptodactylus mystacinus* (Leptodactylidae; [59]). During the leading steps of the courtship, *Ameerega braccata* and species of *Aplastodiscus*, for example, show the same mate positioning that we observed for *H*. *japi*. Studies exploring potential complexities in intraspecific communication of Neotropical frog species are appropriate, especially concerning multimodal compositions.

From 46 species currently recognized for the family Hylodidae [60], ten species are known to perform visual signals (including the species studied in the present work; Table 3). There are data on visual communication for two *Crossodactylus* and eight *Hylodes* species, and no information on *Megaelosia* communication. It is reasonable to believe that the gap in data concerning communication of hylodids is a consequence (at least partial) of their wary and secretive behaviors, making studies difficult, be it in the field or in captivity ([2, 61]; present study).

**Table 3:** Diversity of intraspecific visual communication in Neotropical torrent frogs (Anura, Hylodidae).

Visual displays	*Crossodactylus gaudichaudii* ^A^	*Crossodactylus schmidti* ^B^	*Hylodes asper* ^C^	*Hylodes cardosoi* ^D^	*Hylodes dactylocinus* ^E^	*Hylodes nasus* ^F^	*Hylodes heyeri* ^G^	*Hylodes japi* ^H^	*Hylodes perere* ^I^	*Hylodes phyllodes* ^J^
**Toe trembling**	-	Males	Males	Males	Males	Males	Males	Males	Males	-
**Toe flagging**	-	Females/Males	Males	-	-	-	Males	Males	-	Males
**Toes posture**	-	-	-	-	Males	-	Males	Males	-	-
**Hind foot lifting**	Males	-	-	-	-	-	-	-	-	-
**Limb shaking**	-	-	Males	-	Males	-	-	Males	-	-
**Leg kicking**	-	Males	-	Males	-	-	-	-	-	-
**Both legs kicking**	-	Males	-	-	-	-	-	-	-	-
**Leg stretching**	Males	-	Females/Males	Males	Males	Males	Males	Males	Males	Males
**Foot flagging**	-	-	Males	Females/Males	Males	Males	-	Males	-	Males
**Limb lifting**	-	Females/Males	Females/Males	Males	Males	-	Males	Females/Males	-	Males
**Arm waving**	Males	-	-	-	-	Males	-	Females/Males	-	-
**Body lowering**	-	Males	-	-	-	-	Males	Males	-	Males
**Upright posture**	-	Females/Males	-	-	-	Males	Males	Males	-	Males
**Two-armed impulse**	-	-	-	-	-	-	-	Males	-	-
**Head bobbing**	-	-	-	-	-	-	-	Males	-	-
**Throat display**	-	-	-	-	-	-	Males	Males	-	Males
**Head snaking**	-	-	-	-	-	-	-	Males	-	Males
**Mouth gaping**	-	-	-	-	-	-	Males	-	-	Males
**Body raising**	-	-	Males	Males	-	-	-	Males	-	Males
**Back raising**	-	-	-	-	Males	-	-	-	-	-
**Body jerking**	Males	Females/Males	-	-	-	-	-	Females/Males	-	-
**Truncated walking**	-	-	-	-	-	-	-	Males	-	-
**Jump display**	-	-	-	-	-	-	Males	Males	-	Males
**Running-jumping display**	-	Females/Males	-	-	-	-	-	-	-	-
**Female displays (*N*)**	0	5	2	1	0	0	0	3	0	0
**Male displays (*N*)**	4	9	7	6	7	5	10	18	2	11
**Total (*N*)**	4	9	7	6	7	5	10	18	2	11

All displays considered here were recorded during intraspecific communication in advertisement, agonistic, and courtship contexts (see text for definitions). Reference: A. [2, 38, 62]; B. [37]; C. [2, 6, 21, 63–65]; D. [24, 66]; E. [2, 25, 61, 65, 67]; F. [38, 67]; G. [33, 65]; H. present study; I. [68]; J. [21, 34].

Among hylodids, the currently known repertoire of visual displays is most complex in *H*. *japi* (Table 3). In fact, *Hylodes japi* has one of the most diverse repertoires of visual displays known within the order Anura. The five new visual displays that we described and categorized here correspond to 20.8% of the visual displays recognized for members of the family Hylodidae and around 13.5% for anurans. We trust that our results on visual communication are not an exception among hylodids (and anurans in general), but a consequence of the time invested to understand the behaviors. Among hylodids, the most studied species have more diverse repertoires, such as *C*. *schmidti*, *H*. *phyllodes*, and *H*. *japi* ([21, 34, 37]; present study; see Table 3). For *Hylodes* species, some behaviors, such as arm lifting and arm waving, are only distinguishable via video analysis. Moreover, other visual displays are performed only during specific situations, making them difficult to observe (because they are rarely executed). Head snaking, for example, was recorded only twice among all studies on hylodids; during courtship, once in *H*. *phyllodes* [34] and in the present study. The accepted male is the only individual that performs head snaking and only during courtship. From the set of information presented here, it is plausible to expect that the complexity observed in the visual communication of *H*. *japi* is similarly widespread within the family Hylodidae, or at least among *Hylodes* species. Complexity of the visual communication system may be a pattern for the Brazilian torrent frogs (*Hylodes* species), and most likely as a phylogenetic trait of the genus. Neotropical torrent frogs (i.e. hylodids) still deserve attention, since new studies on their communication have potential to help clarify behavioral patterns and multimodal compositions, and even uncover other new behaviors. Behavioral patterns tend to be similar within families and within genera [2].

The shift to diurnal activity facilitated the evolution of visual communication in frogs [2]. Authors have suggested that visual repertoires seem to be more complex in species that breed at noisy streams and even more complex in species that breed and feed at the same terrestrial sites. They also suggest that future investigations of less-studied species could reveal a distinct scenario. Indeed, even with several new records of visual displays being performed by different species, the superfamily Dendrobatoidea (aromobatids and dendrobatids) still exhibits one of the most complex visual communication systems among frogs. However, comparatively, hylodids are starting to exhibit an even more elevated level of complexity, as observed in the repertoires of *H*. *heyeri*, *H*. *phyllodes*, and *H*. *japi*.

It is conceivable to expect the occurrence of complex visual communication modulated by the environment in fast-flowing stream dwelling diurnal frogs, such as hylodids (e.g., [6, 25, 37, 53]). In comparison with Brazilian torrent frogs (*Hylodes* species), other diurnal torrent frogs in the world, such as Indian frogs *Micrixalus* species (Micrixalidae; [19, 22, 69–71]) and Bornean frogs *Staurois* species (Ranidae; [18, 23, 72, 73]), present similar visual displays. These three tropical genera (*Hylodes*, *Micrixalus*, and *Staurois*) share similarities in breeding habitats, daytime breeding habits, reproductive modes, conspicuous visual displays (e.g., foot flagging), vast repertoires of visual displays, and multimodal communication (e.g., see [22, 69–73]). Since *Hylodes*, *Micrixalus*, and *Staurois* are from distinct phylogenetic groups [74–76] and distinct parts of the world, their behavioral similarities most likely are convergences due to similar ecological pressures. However, it is hard to do any kind of statement about homologies given the lack of behavioral knowledge for several intermediate linages. Overall, frog communication seems to be more complex in tropical regions, probably due to the greater number of species, phylogenetic groups, and/or ecological factors (such as higher microhabitat diversity).

Lastly, in recent years, new windows have been opened concerning the evolution of communication in frogs by the study of other fascinating communication modes, which have been uncovered. For example, water wave communication in the basal frogs of the genus *Bombina* [77, 78], chemical communication in the basal species *Leiopelma hamiltoni* [79, 80], pure ultrasonic communication in the frog *Huia cavitympanum*, the first record for a non-mammalian vertebrate [81], and vibrational communication in the arboreal frog *Agalychnis callidryas* [82]. Among frogs, all of these new communication channels mentioned above, as well as the control and directionality of the vocal sacs (such as observed in the present study), and even new integration between distinct communication modes (such as visual-tactile bimodal signaling in *H*. *japi* females, also observed in the present study), are improving our comprehension on the diversity, complexity, sophistication, and evolution of communication in anurans considerably; a vast field remaining to be investigated.

## Supporting Information

S1 Calls Audio showing *Hylodes japi* males executing advertisement, peep, and squeal calls (advertisement and agonistic contexts). Audio is deposited at the Célio F. B. Haddad sound collection (CFBH), located in Departamento de Zoologia, Instituto de Biociências of Universidade Estadual Paulista (UNESP), Rio Claro, São Paulo, Brazil. Calls recorded on 15–17 April 2011. For all calls, air temperature 24°C and water temperature 19.5°C.
(WAV)

S1 Movie *Hylodes japi* male experimenting with new positions in order to perform undistorted advertisement calls. Male seems to perceive his distorted calls being produced when his inflated vocal sacs touch the water surface. He experiments with new positions to call out of the water. Apparently, the male looks for better positions, until producing the regular call. Recorded on 12 April 2011.
(MP4)

S2 Movie *Hylodes japi* male calling and performing visual displays in long-range agonistic context. Male emits advertisement, peep, and squeal calls. Advertisement calls are always emitted by using both vocal sacs. Peep and squeal calls can be emitted with the use of both vocal sacs simultaneously or only with a single vocal sac. While calling, male performs visual displays (arm lifting, arm waving, head bobbing, body jerking, and truncated walking). Slowing the movie down (rate 0.25), we can better observe the execution of arm waving, with the male’s hand passing in front of the whitish-silver area of his head. Recorded on 8 March 2012.
(MP4)

S3 Movie *Hylodes japi* male calling and performing visual displays with hind limbs in long-range agonistic context. Male performs toe trembling, toe flagging, toes posture, and foot flagging, while emitting advertisement calls. Recorded on 8 March 2012.
(MP4)

S4 Movie Evidence of the visual functions of vocal sacs in *Hylodes japi* males. (I) In long and short-range agonistics contexts, the movie shows males executing throat displays (pulsating the vocal sacs) alternating with peep and squeal calls preceding advertisement calls, or combined with another visual display (e.g., foot flagging). Recorded on 7–8 March 2012. (II) In a short-range agonistic context, resident male emits peep and squeal calls, with vocal sac inflation directed toward a conspecific intruder male (which is in body lowering posture). Recorded on 26 March 2011.
(MP4)

S5 Movie *Hylodes japi* couple during the elaborate courtship ritual. Movie starts showing female approaching the male and the exact moment when the female adopts the acceptance position, touching the male’s dorsum with her gular region. That couple position is kept during the acceptance courtship step. Then, we can observe the male emitting peep and squeal calls with only one vocal sac inflated, the one nearer the female (his left vocal sac), demonstrating the visual component of his bright whitish vocal sacs. Next, we can observe two events whereby the female triggered the male’s courtship calls via bimodal stimulation (visual plus tactile); first by arm lifting display plus tactile stimulation and second by arm waving display plus tactile stimulation. The movie is slowed down for better observation (rate 0.25). Finally, once male and female reach the fast-flowing stream margin, we can see the exact moment when, while maintaining physical contact with the female, the male slightly moves his body forward, consequently moving the female body as well; then the male dives followed by the female, marking the beginning of the underwater part of the courtship (leading to the oviposition site). Recorded on 6 March 2012, using the camera’s ‘NightShot’ function (infrared sensitivity).
(MP4)
